# UVA Light Triggers Activation of TRPV1 and TRPA1 by Staurosporine and Midostaurin

**DOI:** 10.3390/ijms27010227

**Published:** 2025-12-25

**Authors:** Sebastian Pantke, Lucas H. K. Weber, Frank G. Echtermeyer, Christine Herzog, Mirjam J. Eberhardt, Andreas Leffler

**Affiliations:** 1 Department of Anesthesiology and Intensive Care Medicine, Hannover Medical School, 30625 Hannover, Germany; 2 PRACTIS Clinician Scientist Program, Dean’s Office for Academic Career Development, Hannover Medical School, 30625 Hannover, Germany

**Keywords:** TRP channel, oxidative stress, photosensitivity, midostaurin, staurosporine

## Abstract

The activation of TRPV1 and TRPA1 by UVA light is a complex process involving channel modulation by reactive oxygen species (ROS). The present study describes staurosporine and midostaurin, two protein kinase inhibitors, as photosensitizers that can modulate the activity of TRPV1 and TRPA1 in a UVA light-dependent manner. Patch-clamp and calcium imaging were used to investigate effects of staurosporine and midostaurin on recombinant human (h) TRPV1 and TRPA1 in HEK 293T cells and on native mouse dorsal root ganglion (DRG) cells. Staurosporine applied alone did not induce channel activation, but co-application with UVA light activated both TRPV1 and TRPA1. Staurosporine with UVA light also potentiated TRPV1-mediated membrane currents induced by heat and protons. Midostaurin induced the UVA light-independent activation and sensitization of TRPV1 and TRPA1, and this effect was strongly potentiated by UVA light. Effects induced by both staurosporine and midostaurin were reversed by the reducing agent dithiothreitol (DTT). Midostaurin induced a calcium influx in TRPA1-expressing DRG neurons. Our results show that staurosporine and midostaurin modulate the activity of TRPV1 and TRPA1 channels in the presence of UVA light. These photosensitizing properties can be relevant when staurosporine is used for in vitro experiments, and they may account for the phototoxic side effects of midostaurin.

## 1. Introduction

A significant number of photosensitizers—molecules that generate reactive oxygen species (ROS) upon absorption of light—are currently in use within the field of medicine. When combined with ultraviolet (UV) light, these compounds can trigger phototoxic or photoallergic reactions following systemic or topical application [1]. Clinically, such reactions are characterized by erythema accompanied by pruritus, burning, or stinging sensations [2]. TRPA1 and TRPV1 represent two members of the heterogeneous group of the transient receptor potential (TRP) channels. TRPA1 and TRPV1 are both non-selective cation channels that are activated by several physical (heat or cold) and chemical stimuli [3]. It is evident that, due to their high expression in sensory neurons, they play a pivotal role in the perception of painful or irritating external stimuli [4]. Importantly, both channels are sensitive to ROS, and their activity can be modulated in phototoxic reactions induced by endogenous or exogenous substances [5,6,7,8].

In phototoxic or photoallergic reactions, photosensitive molecules can absorb light and are then transformed into an unstable, energy-rich singlet state. The return to a more stable state releases energy, which can participate directly or indirectly in cellular reactions through ROS formation. The ROS-sensitivities of TRPA1 and TRPV1 are primarily attributable to the modification of specific intracellular cysteine residues [9,10,11].

Previous studies from our laboratory and others have suggested that kinase inhibitors can unexpectedly modulate TRP channel activity. Staurosporine, a competitive kinase inhibitor widely used in research, has been shown to enhance heat-induced currents mediated by TRPV1 [12]. Given that the activation of protein kinases A and C typically promotes TRPV1 activity [13,14], this effect was surprising. Notably, Lee et al. employed staurosporine as a PKC inhibitor in studies of UV light-induced activation of HaCaT cells, illustrating its complex cellular effects [15].

While staurosporine is not clinically used due to its broad kinase-binding profile [16], its semi-synthetic derivative, midostaurin, is approved for the treatment of acute myeloid leukemia and mastocytosis [17]. Midostaurin is a multitarget kinase inhibitor, and a case report described a photoallergic reaction in a patient following midostaurin intake [18,19]. This observation may highlight its potential for light-dependent effects. Taken together, these observations raise the question of whether staurosporine and midostaurin can induce UVA light-dependent modulation of TRPA1 and TRPV1.

The present study aims to evaluate the effects of these kinase inhibitors on TRP channel activity under UVA light exposure. Our data provide insights into the mechanisms underlying photosensitizer-induced sensory reactions.

## 2. Results

### 2.1. Staurosporine Activates hTRPA1 in a UVA Light-Dependent Manner

**Figure 1:**
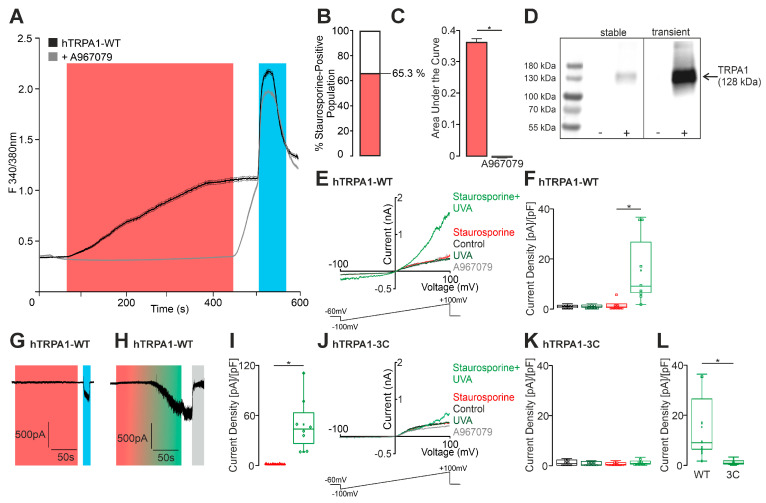
Staurosporine activates hTRPA1 in a UVA light-dependent manner. (**A**) Ratiometric calcium imaging of hTRPA1-expressing HEK293T cells showing the effect of staurosporine (100 nM, 360 s; red bar). The black trace represents the staurosporine condition (*n* = 1197, examined in 10 independent experiments), while the gray trace (*n* = 1037, examined in 10 independent experiments) shows the same protocol with concomitant application of the TRPA1 antagonist A967079. Carvacrol (300 µM, 60 s; blue bar) served as a positive control. Data are presented as mean ± S.E.M (shown as thin shaded traces). (**B**) Stacked bar chart summarizing the total population of hTRPA1-expressing HEK293T cells analyzed. The red portion indicates the fraction of cells responding to staurosporine. (**C**) Areas under the curve (AUCs) of staurosporine-evoked calcium responses (red) compared with co-application of A967079 (gray). Error bars represent S.E.M corresponding to data in panel **A** (ANOVA followed by Tukey’s HSD post hoc test). (**D**) Western blot analysis demonstrating TRPA1 expression in stably expressing HEK293 cells compared to parental HEK293 cells. TRPA1 expression in transiently transfected HEK293T cells is shown alongside the negative control (untransfected HEK293T). (**E**) Whole-cell patch clamp recordings using 500 ms voltage ramps from −100 to +100 mV during exposure to UVA light (dark green), staurosporine (100 nM; red trace, *n* = 7), and combined staurosporine + UVA light (green trace, *n* = 7). Each application lasted for 20 s. Mean capacitance: 12.9 ± 1.6 pF, mean access resistance: 8.7 ± 1.5 MΩs. (**F**) Current densities at −60 mV presented as box-and-scatter plots. A significant increase in current density was observed with the combined application of staurosporine and UVA light (*n* = 7, ANOVA with Tukey’s post hoc test). (**G**–**I**) Whole-cell patch clamp recordings of hTRPA1-expressing HEK293T cells at a holding potential of −60 mV with or without UVA light exposure. Mean capacitance: 13.9 ± 1.6 pF, mean access resistance: 7.5 ± 1.1 MΩ. (**G**) Staurosporine (100 nM, 120 s; red bar, *n* = 9) was applied before administration of carvacrol (300 µM; blue bar). (**H**) The experiment was repeated under UVA light illumination (red–green bar, *n* = 8). A967079 (100 nM; gray bar) was applied at the end of each experiment. (**I**) Box-and-scatter plot showing current densities for experiments in panels **G** and **H**, demonstrating a significant effect of UVA light on staurosporine responses (unpaired *t*-test). (**J**,**K**) The experiment shown in panel **E** was repeated using the ROS-insensitive TRPA1-3C mutant (*n* = 8). Mean capacitance: 16.8 ± 2.3 pF, mean access resistance: 8 ± 1.1 MΩ. (**L**) Box-and-scatter plot summarizing the responses of the mutant channel, demonstrating a significant reduction in staurosporine + UVA light-evoked currents compared with wild-type TRPA1 (unpaired *t*-test). * denotes *p* < 0.05. The box denotes the 50th percentile (median) as well as the 25th and 75th percentiles. The whiskers mark the 5th and 95 percentiles. Data points beyond the whiskers are outliers.

To determine if staurosporine modulates TRPA1, ratiometric calcium imaging was performed in HEK 293 cells stably expressing hTRPA1. The application of staurosporine (100 nM) indeed induced a robust increase in intracellular calcium (Figure 1A), and this effect was completely blocked by co-application of the selective TRPA1-inhibitor A967079 (10 µM, Figure 1A–C). Functional TRPA1 expression was verified by application of the TRPA1 agonist carvacrol (300 µM). TRPA1 expression was also verified by Western blot analyses, showing that TRPA1 was strongly expressed in stable TRPA1-HEK293 cells, and was even stronger in HEK 293T following transient transfection (Figure 1D). To corroborate TRPA1 activation by staurosporine, whole-cell patch clamp experiments were performed. The expression of hTRPA1 was again verified by carvacrol (300 µM), and the antagonist A967079 (100 nM) was used to confirm TRPA1-specific activation. 500 ms long voltage ramps from −100 to +100 mV were applied, which remained unchanged by the application of staurosporine or UVA light (Figure 1E). However, these ramp currents were potentiated when UVA light and staurosporine were co-applied (Figure 1E,F). Similar effects could also be observed when cells were held at −60 mV. While the sole application of staurosporine did not evoke any inward currents (Figure 1G), its co-application with UVA light resulted in large inward currents that were fully inhibited (e.g., 100%) by A967079 (Figure 1H,I). Therefore, staurosporine appears to exhibit characteristics of a photosensitizer that can induce activation of TRPA1 in a UVA light-dependent manner. In order to validate this understanding, we examined the ROS-insensitive mutant hTRPA1 C621S/C641S/C665S [9]. Indeed, co-application of staurosporine and UVA light did not result in an augmentation of inward currents in cells expressing this mutant (Figure 1J,K). These data support the concept of the activation of TRPA1 by ROS generated by staurosporine in the presence of UVA light (Figure 1L).

### 2.2. Staurosporine Sensitizes hTRPV1 by Producing ROS

**Figure 2:**
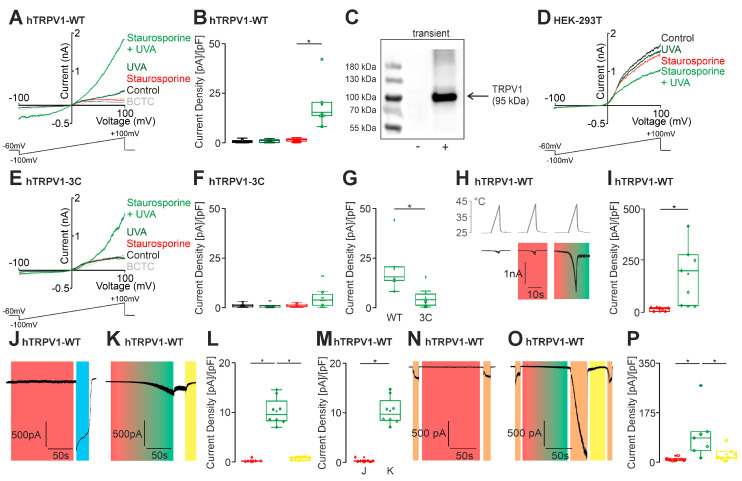
Staurosporine sensitizes hTRPV1 via ROS production. (**A**) Whole-cell recordings from hTRPV1-expressing HEK293T cells using voltage ramps from −100 to +100 mV. Cells were stimulated with UVA light alone (dark green), staurosporine (100 nM) alone (red trace, *n* = 8), or the combined application of staurosporine and UVA light (green trace, *n* = 8). Mean capacitance: 14.9 ± 2.1 pF, mean access resistance: 8.4 ± 1.6 MΩ. (**B**) Box-and-scatter plots of current densities at −60 mV corresponding to panel **A**. A significant increase in current density was observed with the combined staurosporine + UVA light stimulation (*n* = 8, ANOVA with Tukey’s post hoc test). (**C**) Western blot showing TRPV1 expression in transiently transfected HEK293T cells compared to untransfected HEK293T controls. (**D**) Voltage ramps (−100 to +100 mV) in parental HEK293T cells showing no changes in membrane currents upon administration of staurosporine and/or UVA light, using the same protocol as in panel **A**. Mean capacitance: 25.2 ± 2.6 pF, mean access resistance: 6.7 ± 1.2 MΩ. (**E**,**F**) The experiment described in panel **A** was repeated using the TRPV1-C158A/C767S/C387S mutant (*n* = 8). Mean capacitance: 15.5 ± 0.6 pF, mean access resistance: 7.9 ± 1.3 MΩ. (**F**) Box-and-scatter plots show no significant activation of the TRPV1-3C mutant by the combined application of staurosporine and UVA light (ANOVA with Tukey’s post hoc test). (**G**) Comparison of wild-type and TRPV1-3C mutant responses demonstrates a significant reduction in staurosporine + UVA light-evoked currents in the mutant channel (unpaired *t*-test). (**H**) Representative heat-ramp traces showing UVA light-dependent activation of hTRPV1 in the presence of staurosporine (red bar, *n* = 7). Staurosporine and UVA light were co-applied for 120 s (red-green bar, *n* = 7). Mean capacitance: 12.6 ± 2.1 pF, mean access resistance: 4.9 ± 0.6 MΩ. (**I**) Box-and-scatter plots corresponding to panel **H** (paired *t*-test). (**J**–**M**) Significant inward currents were observed during combined staurosporine + UVA-ligth application at a holding potential of –60 mV. Mean capacitance: 21.8 ± 4.2 pF, mean access resistance: 6.8 ± 0.9 MΩ−60 mV. (**J**) Staurosporine alone (100 nM, 120 s; red bar, *n* = 8). As a positive control the specific agonist capsaicin was applied (1 µM, 20 s, blue bar). (**K**) Same protocol with concurrent UVA light exposure (red-green bar); DTT was applied at the end of the protocol (yellow bar, *n* = 8). (**L**) Box-and-scatter plots of current densities corresponding to panel **K** (ANOVA with Tukey’s post hoc test). (**M**) Box-and-scatter plot of current densities from panels **J** and **K** (unpaired *t*-test). (**N**–**P**) Co-application of UVA light and staurosporine enhanced proton-evoked currents, an effect reversed by DTT. Mean capacitance: 17.5 ± 2.6 pF, mean access resistance: 7.4 ± 1.4 MΩ. (**N**) Staurosporine (red bar, 120 s) during repeated pH-evoked responses (10 s applications, pH 6.2; orange bars, *n* = 8). (**O**) Sensitization of pH-evoked currents during combined staurosporine + UVA light application (red-green bar, 90 s), which was prevented by DTT (yellow bar, 60 s; *n* = 8). (**P**) Box-and-scatter plots of current densities corresponding to panels **M** and **N** (ANOVA with Tukey’s post hoc test). * denotes *p* < 0.05. The box denotes the 50th percentile (median) as well as the 25th and 75th percentiles. The whiskers mark the 5th and 95 percentiles. Data points beyond the whiskers are outliers.

Photosensitizers can also activate and sensitize TRPV1 [6,7,8]. We therefore asked if staurosporine could be considered a photosensitizer in relation to TRPV1. We performed patch clamp experiments on HEK 293T cells expressing TRPV1 and found that application of 100 nM staurosporine did not induce any membrane currents (Figure 2A). However, the co-application of staurosporine and UVA light resulted in a robust outwardly rectifying membrane current (Figure 2A,B). The expression of TRPV1 was verified by application of the TRPV1 inhibitor BCTC at the end of the experiment, and also by Western blots demonstrating a strong expression of TRPV1 (Figure 2C). Furthermore, the same protocol did not generate currents in native HEK cells (Figure 2D). In order to verify the influence of ROS in this instance as well, we performed this protocol on the TRPV1-C158A/C767S/C387S mutant (Figure 2E) [20]. These measurements revealed a significant reduction in the effect of staurosporine and UVA light, but the effect was not completely eliminated (Figure 2F,G). This remaining ROS sensitivity of TRPV1-C158A/C767S/C387S was observed in a previous report [12]. We also observed a UVA light dependent sensitization of heat-evoked currents upon co-application of staurosporine (Figure 2H,I). Robust inward currents were observed in response to the concurrent application of UVA-light and staurosporine, but not to the sole application of staurosporine (Figure 2J–M). As a positive control capsaicin (1 µM) was applied. The concomitant application of UVA light and staurosporine also resulted in a potentiation of proton-evoked currents (pH 6) (Figure 2N–P). To verify that the observed effect is attributable to ROS-production, patch-clamp experiments were performed with the reduction agent dithiotreitol (DTT 10 mM). Indeed, membrane currents induced by staurosporine and UVA light were reversed by DTT (Figure 2K,L). Furthermore, the application of 10 mM DTT effectively prevented the sensitization of hTRPV1 to protons (Figure 2O,P). These data show that staurosporine can activate the ROS-sensitive TRP channels TRPA1 and TRPV1 via production of ROS, when it is illuminated with UVA light.

### 2.3. Midostaurin Activates hTRPV1

**Figure 3:**
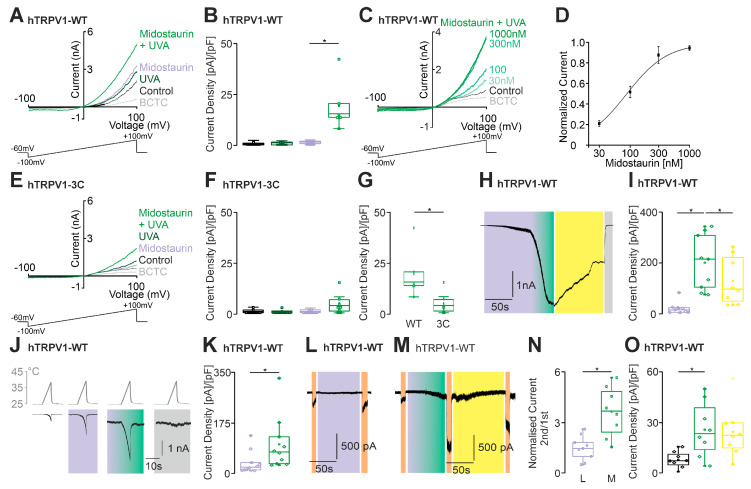
Midostaurin activates and sensitizes hTRPV1. (**A**) Whole-cell voltage–clamp recordings from hTRPV1-expressing HEK293T cells using voltage ramps from −100 to +100 mV. Cells were stimulated with UVA light alone (dark green), midostaurin (100 nM) alone (purple, *n* = 8), or midostaurin together with UVA light (green, *n* = 8). (**B**) Box-and-scatter plots of current densities at −60 mV corresponding to panel **A**. A significant increase in current density was observed for the combined midostaurin + UVA condition (ANOVA with Tukey’s post hoc test). (**C**) Increasing concentrations of midostaurin were applied during UVA light illumination (green traces; darker saturation indicates higher concentration, *n* = 8). The TRPV1 blocker BCTC (100 nM) was applied at the end (gray trace). (**D**) Concentration–response curve for midostaurin-induced activation of hTRPV1. Current amplitudes at +100 mV were normalized to the response evoked by 1000 µM midostaurin. The fitted curve represents a Hill model. (**E**–**G**) The experiment in panel **A** was repeated using the TRPV1-3C mutant (*n* = 6). (**F**) Box-and-scatter plots show no significant activation of the TRPV1-3C mutant by midostaurin + UVA light (ANOVA with Tukey’s post hoc test). (**G**) Comparison of wild-type and TRPV1-3C mutant responses reveals a significant reduction in midostaurin + UVA light-induced currents in the mutant (unpaired *t*-test). (**H**) Representative current trace showing UVA-dependent sensitization of midostaurin-induced currents (purple-green bar). DTT partially reversed the sensitization (yellow), and BCTC (100 nM; gry bar) completely inhibited the response (*n* = 9). (**I**) Box-and-scatter plots of current densities corresponding to panel **H** (ANOVA with Tukey’s post hoc test). (**J**,**K**) Heat-evoked currents in hTRPV1-expressing cells showing potentiation by midostaurin alone (purple bar) and by midostaurin + UVA light (purple–green bar, *n* = 10 each). BCTC (100 nM) abolished heat-evoked responses (gray). (**K**) Box-and-scatter plots corresponding to panel **J** (paired *t*-test). (**L**,**M**) Midostaurin alone (purple bar, 100 nM) or midostaurin + UVA light (purple-green bar) sensitized pH-evoked currents (pH 6.2; 10 s pulses, orange bars). Sensitization was partially reversible by DTT (10 mM; yellowbar, *n* = 9). (**N**) Box-and-scatter plots showing normalized pH-evoked currents corresponding to panels **L** and **M**. Second pH responses were normalized to the first pH pulse (unpaired *t*-test). (**O**) Box-and-scatter plots of current densities from the experiments shown in panel **M** (ANOVA with Tukey’s post hoc test). * denotes *p* < 0.05. The box denotes the 50th percentile (median) as well as the 25th and 75th percentiles. The whiskers mark the 5th and 95 percentiles. Data points beyond the whiskers are outliers.

Midostaurin has been associated with photosensitivity reactions in case reports [18,21]. We first examined the effects of midostaurin on cells expressing hTRPV1. In contrast to staurosporine, midostaurin itself induced outwardly rectifying currents in hTRPV1-expressing cells (Figure 3A). When UVA light was added to midostaurin, large membrane currents were induced that were inhibited by the TRPV1-inhibitor BCTC (Figure 3A,B). This midostaurin-induced effect displayed a clear concentration dependency, with an EC_50_-value of 187 ± 14 µM (Figure 3C) measured at +100mV. In order to confirm a ROS-mediated effect for midostaurin as well, the ROS-insensitive mutant TRPV1-C158A/C767S/C387S was utilized at this point (Figure 3E). In accordance with the data from staurosporine, this continued to demonstrate a slight potentiation in a co-application with midostaurin and UVA light in outwardly rectifying currents, but overall, a significant reduction in current increase was observed (Figure 3F,G).

In cells held at −60 mV, the concomitant application of midostaurin and UVA light generated large inward currents that were partly reversed by DTT (Figure 3H,I). We also observed an increase in heat-evoked currents (Figure 3J,K) as well as proton-evoked currents (pH6) (Figure 3L–O) when midostaurin and UVA light were applied. Our findings suggest that ROS might be crucial for the activation of hTRPV1 channels by midostaurin and UVA light.

### 2.4. Midostaurin Activates hTRPA1

**Figure 4:**
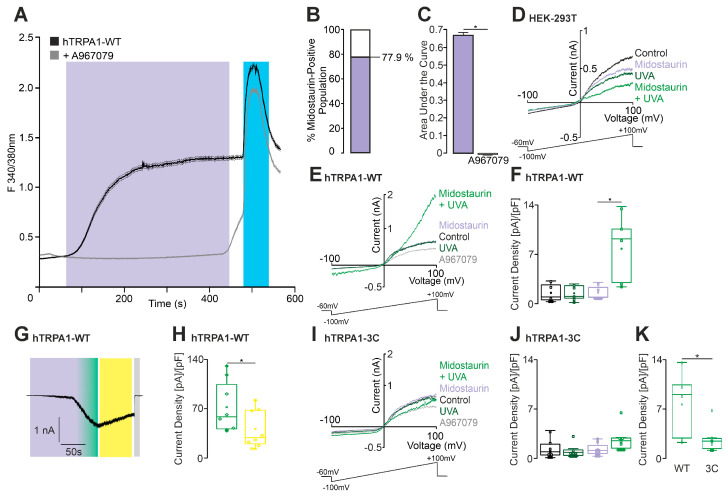
Midostaurin activates hTRPA1. (**A**) Ratiometric calcium imaging of hTRPA1-expressing HEK293T cells showing the effect of midostaurin (100 nM, 360 s; purple bar). The black trace shows the midostaurin condition (*n* = 839, examined in eight independent experiments), while the gray trace (*n* = 996, examined in eight independent experiments) represents the same protocol with concomitant application of the TRPA1-antagonist A967079. Carvacrol (300 µM, 60 s; blue bar) served as a positive control. Data are presented as mean ± S.E.M., shown as thin shaded traces. (**B**) Stacked bar chart summarizing the total hTRPA1-expressing HEK293T cell population analyzed. The purple portion indicates the fraction of cells responding to midostaurin. (**C**) Areas under the curve (AUCs) of midostaurin-evoked calcium signals (purple) compared with co-application of A967079 (gray). Error bars represent S.E.M. (ANOVA followed by Tukey’s HSD post hoc test; corresponds to data in panel **A**). (**D**) Voltage ramps (−100 to +100 mV) in parental HEK293T cells showing no change in membrane currents during application of midostaurin and/or UVA light. Mean capacitance: 18.1 ± 3.8 pF, mean access resistance: 6.2 ± 0.2 MΩ. (**E**) Whole-cell recordings from hTRPA1-expressing HEK293T cells using voltage ramps from −100 to +100 mV. Cells were stimulated with UVA light alone (dark green), midostaurin (100 nM) alone (purple, *n* = 8), or midostaurin together with UVA light (green, *n* = 8). Mean capacitance: 14.5 ± 2 pF, mean access resistance: 8.1 ± 0.7 MΩ. (**F**) Box-and-scatter plots showing current densities at −60 mV, corresponding to panel **E**. The combination of midostaurin + UVA light elicited a significant increase in current density (ANOVA with Tukey’s post hoc test). (**G**) Representative current trace showing potentiation of midostaurin-evoked inward currents by UVA light. Midostaurin (100 nM) was applied for 80 s (purple bar), followed by combined midostaurin + UVA light for 50 s (purple-green bar). Sensitization was partially reversible by DTT (10 mM; yellow bar). A967079 (100 nM; gray bar, *n* = 8) inhibited the response. Mean capacitance: 12.4 ± 1.2 pF, mean access resistance: 4.1 ± 0.9 MΩ. (**H**) Box-and-scatter plots showing current densities from panel **G**, demonstrating a significant effect of DTT on midostaurin + UVA light responses (paired *t*-test). (**I**,**J**) The experiment in panel **E** was repeated on the TRPA1-3C mutant (*n* = 6). Mean capacitance: 20.5 ± 2.1 pF, mean access resistance: 8.9 ± 1.4 MΩ. (**J**) Box-and-scatter plots show no significant activation of the TRPA1-3C mutant by midostaurin + UVA light (ANOVA with Tukey’s post hoc test). (**K**) Comparison of wild-type and TRPA1-3C mutant responses shows a significant reduction in midostaurin + UVA light-evoked currents in the mutant (unpaired *t*-test). * denotes *p* < 0.05. The box denotes the 50th percentile (median) as well as the 25th and 75th percentiles. The whiskers mark the 5th and 95 percentiles. Data points beyond the whiskers are outliers.

Ratiometric calcium imaging was performed on HEK 293 cells stably expressing hTRPA1 to investigate the effects of midostaurin. Indeed, midostaurin induces a significant influx of calcium in TRPA1-expressing cells. This effect was inhibited by the specific TRPA1-blocker A967079 (Figure 4A,C). Midostaurine did not evoke any UVA light-dependent membrane currents in native HEK 293T cells (Figure 4D). In hTRPA1-expressing cells, however, midostaurin induced outwardly rectifying currents when applied with UVA light (Figure 4E,F). DTT partially reversed this effect (Figure 4G,H). These findings suggest that midostaurin activates TRPA1 in a UVA light-dependent manner and that ROS plays a crucial role in this process. This hypothesis was confirmed through experimentation with the ROS-insensitive TRPA1-3C mutant, which revealed a substantial decline in the inward current at −60 mV (Figure 4I–K).

### 2.5. Midostaurin Activates TRPA1-Expressing Mouse DRG Neurons

**Figure 5:**
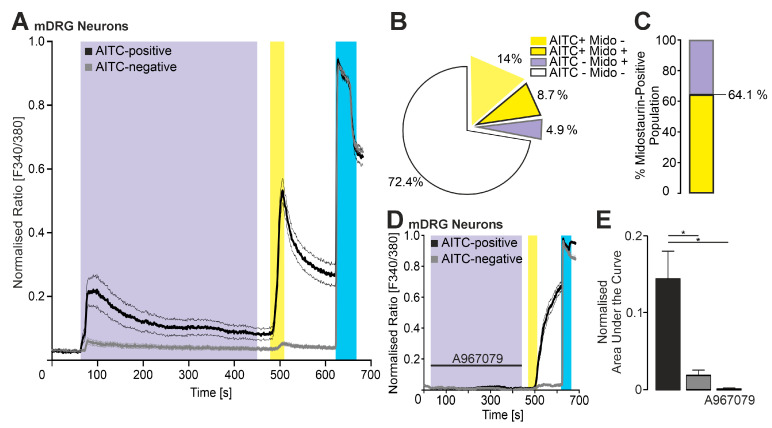
Midostaurin activates mouse DRG neurons in a TRPA1-dependent manner. (**A**) Ratiometric calcium imaging showing midostaurin-induced increase in intracellular calcium (100 nM, 360 s; purple bar) in mouse DRG neurons (mDRGs). Neurons were classified based on their response to the TRPA1 agonist AITC (100 µM, 20 s; yellow bar) in TRPA1-expressing mDRGs (*n* = 65 cells in total, black trace) and non-TRPA1-expressing mDRGs (*n* = 221, from 7 independent measurements). Potassium chloride (60 mM, 60 s; blue bar) was applied as a positive control. (**B**) Pie chart summarizing the total population of mDRGs responding to AITC and/or midostaurine or irresponsive to both. (**C**) Stacked bar chart showing the whole proportion of midostaurine-responsive DRG neurons sorted by responsiveness to AITC. (**D**) Coapplication of midostaurine and the TRPA1-selective antagonist A967079 verifies TRPA1-dependent activation of mouse DRG neurons by midostaurin (black: AITC-positive mDRGs, *n* = 208; gray: AITC-negative mDRGs, *n* = 535, from 4 independent measurements). (**E**) Quantification of normalized areas under the curve (AUC) for midostaurin-evoked calcium responses under different conditions: AITC-positive neurons (black), AITC-negative neurons (gray), and midostaurin + A967079 (purple/gray). Error bars represent S.E.M. (ANOVA followed by Tukey’s HSD post hoc test; corresponds to data in panel **A**). * denotes *p* < 0.05. The box denotes the 50th percentile (median) as well as the 25th and 75th percentiles. The whiskers mark the 5th and 95 percentiles. Data points beyond the whiskers are outliers.

Finally, we sought to investigate whether Midostaurin (100 nM) activates mouse DRG neurons in a TRPA1-dependent manner. As demonstrated in Figure 5A, ratiometric calcium imaging revealed a substantial increase in intracellular calcium following application of midostaurin. Allylisothiocyanat AITC (100 µM) was applied to determine the TRPA1-expressing population, and KCl 60 mM was used as a final control to ensure viable DRG neurons. AITC-sensitive, i.e., TRPA1-expressing cells exhibited a larger midostaurin-induced increase in intracellular calcium as compared to AITC-negative cells (Figure 5A–C). To prove that the effect of midostaurin on mDRGs is indeed mediated by hTRPA1, coapplication of midostaurin and the TRPA1-specific antagonist A967079 was investigated, which completely blocked midostaurin-induced increase in intracellular calcium in mouse DRG neurons. (Figure 5D,E).

## 3. Discussion

The study of TRPA1 and TRPV1 and their regulation by UVA light has significant implications for the field of photobiology and the understanding of the interactions between light and biological systems [6,7,8]. Our study describes the mechanisms underlying the sensitization of hTRPA1 and hTRPV1 by staurosporine and midostaurin. Our results demonstrate that UVA light enhances the activation of hTRPA1 and hTRPV1 by both substances, a phenomenon that seems to be mediated by the generation of ROS.

A case report by Cura et al. described a patient with systemic mastocytosis who developed a midostaurin-induced lichenoid photoallergic reaction [16]. This case highlights the potential for midostaurin to cause photoallergic reactions in patients. The elucidation of the mechanisms underlying the sensitization of ROS-sensitive TRP channels TRPA1 and TRPV1 by UVA light in the presence of midostaurin can thus have significant clinical implications. A potential explanation for this phenomenon is the inhibition of ABCG2 by midostaurin. ABCG2 is a member of the ATP-binding cassette (ABC) transporters responsible for the transport of a wide range of substrates across cell membranes [22]. Ning Ji et al. demonstrated that midostaurin inhibits members of the ATP-binding cassette transporter family [23], and an inhibition of ABCG2 can result in the accumulation of protoporphyrin IX [24]. Of note, protoporphyrin IX has been shown to activate TRPA1 and TRPV1 via ROS-production when illuminated with UVA light [5]. The present study is the first to demonstrate both ROS-independent and ROS-dependent activation of TRPV1 and TRPA1 by midostaurin. Therefore, it seems plausible that midostaurin itself and the midostaurin-induced accumulation of protoporphyrin IX are relevant for these phototoxic reactions. With that said, the number of reported cases with a confirmed midostaurin-evoked phototoxicity is limited. This may indicate that the clinical relevance is marginal, or that phototoxic reactions in these patients are not interpreted as being a result of the therapy with midostaurin. However, when taking into consideration the prevalence of AML or systemic mastocytosis, for which midostaurin is utilized, the anticipated frequency of cases of photosensitivity among these patients may increase [25]. In contrast to this clinical relevance of midostaurin being a photosensitizer, the relevance for the observed UVA light-dependent modulation of TRPA1 and TRPV1 by staurosporine is limited to its use in basic research. Staurosporine is employed as a competitive kinase inhibitor, and we previously demonstrated that staurosporine can enhance heat-induced currents mediated by TRPV1 [12]. Given that an activation of protein kinases A and C typically sensitizes TRPV1 activity [13,14], this effect from a kinase inhibitor was surprising. Notably, Lee et al. employed staurosporine as a PKC inhibitor in studies on UVA light–induced activation of HaCaT cells [15]. Under experimental conditions where cells are exposed to UVA light, we believe that the interpretation of staurosporine-induced cellular effects should not be understood as the sole result of an inhibition of kinases. Staurosporine is also frequently used for in vitro induction of mitochondrial-mediated apoptosis [22,23]. Considering that UVA light can also be employed to induce apoptosis, the property of staurosporine as a photosensitizer found in this study seems to be relevant in this regard as well.

Our data indicate that the activation of both TRPA1 and TRPV1 by staurosporine or midostaurin + UVA light mainly depends on a modification of intracellular cysteine residues known to dictate the ROS-sensitivity of these channels [15]. This mechanistic principle for the activation of TRPA1 and TRPV1 by photosensitizers was also demonstrated in previous studies [5,6,7,8]. However, our findings align with our previous observations, in that UVA light-induced gating of TRPV1 also involves cysteine-independent mechanisms [12].

While our findings provide important insights into the photosensitizing properties of staurosporine and midostaurin, some methodological limitations should be acknowledged. First, the use of carvacrol as a TRPA1 agonist, although effective in eliciting robust channel activation, is not entirely specific, as it has been reported to activate other TRP channels, including TRPV3 and TRPM8. Although we confirmed the functional expression of TRPA1 through the use of the selective antagonist A967079, which fully reversed carvacrol-induced responses, the possibility of off-target effects cannot be entirely ruled out. Second, the internal pipette solution used in patch clamp experiments contained high concentrations of potassium (140 mM) and chloride (140 mM), which may contribute to significant outward currents under physiological voltage conditions. These currents, primarily carried by K^+^ and Cl^−^ ions, can mask or distort the amplitude and kinetics of TRP channel-mediated currents, especially when recorded in voltage–clamp mode. Although outward currents are expected under depolarized conditions, the large inward currents observed in Figure 2O (hTRPV1-WT) at hyperpolarized potentials (−60 mV) are notable and may reflect the combined contribution of residual endogenous currents or non-specific ion fluxes. Future experiments could consider replacing intracellular K^+^ with cesium (Cs^+^) or intracellular Cl^−^ with gluconate to minimize these confounding ionic contributions and improve the specificity of recorded currents. On the other hand, the large inward currents observed in Figure 2O (hTRPV1-WT) may also be influenced by the overexpression of TRPV1 in HEK293T cells, which can lead to altered channel density, subcellular localization, and increased susceptibility to endogenous modulators. TRPV1 is known to be sensitized by both extracellular protons (low pH) and reactive oxygen species (ROS). In our experimental setup, the combination of high channel expression, local acidification from cellular metabolism, and UVA-induced ROS production may synergistically enhance TRPV1 open probability and current amplitude, particularly at hyperpolarized potentials where the driving force for cations is maximal.

In summary, the present study offers novel insights into the properties of staurosporine and midostaurin as photosensitizers able to activate and sensitize the ROS-sensitive ion channels TRPV1 and TRPA1. While further research is warranted to delineate the underlying mechanisms as well as the clinical relevance of these effects, they should be taken into account when staurosporine is used for in vitro cellular assays.

## 4. Materials and Methods

### 4.1. Chemicals

The chemicals were procured and dissolved in the following manner: Staurosporine was obtained from Alomone Labs (Jerusalem, Israel) and Midostaurin from Sigma-Aldrich (Taufkirchen, Germany). Both substances were diluted in DMSO and stored at −20 °C in light-protected conditions. Dithiotreitol (DTT) from Sigma Aldrich (Taufkirchen, Germany) was dissolved immediately prior to use. The specific channel modulators BCTC, capsaicin, and A967079 were obtained from HelloBio (Bristol, UK). Carvacrol was obtained from Sigma Aldrich (Taufkirchen, Germany). All samples were diluted in DMSO and stored at a temperature of +4 °C.

### 4.2. cDNA and Cell Culture

For the electrophysiological experiments, HEK293T cells (ATCC CRL-3216) were used. The cells were transfected with various constructs of hTRPV1 and hTRPA1 using jetPEI (VWR, Darmstadt, Germany). The cells were cultured in a specialized medium consisting of Dulbecco’s modified Eagle medium nutrient mixture F12 (DMEM/F12, Gibco/Invitrogen, Darmstadt, Germany) supplemented with 10% fetal bovine serum (Biochrom, Berlin, Germany). The experiments were conducted under standard cell culture conditions, with a 5% CO_2_ atmosphere at 37 °C. For the patch clamp experiments, the cells were cultured for approximately 24 h and then detached using phosphate-buffered saline (PBS, Lonza, Cologne, Germany).

### 4.3. Animals

DRG neurons were obtained from C57Bl/6 WT mice as previously described [24]. The experiment involved three adult male mice. All of them were prepared and cultured according to the standard protocol outlined by Sokol et al. by the same person. For the purposes of this study, dorsal root ganglia from all heights were utilized and considered collectively. Mouse DRGs were harvested and processed as follows: animals were anesthetized with isoflurane and euthanized by decapitation. The DRGs were then collected from all spinal column levels and transferred to a DMEM solution. The ganglia were then subjected to enzymatic digestion using a DMEM solution containing collagenase (1 mg/mL) and protease (0.5 mg/mL) for 45 min. The isolated cells were then separated using a Pasteur pipette and plated on poly-L-lysine-coated coverslips. The cells were then cultured in a TNB 100 medium supplemented with a lipid protein complex, penicillin/streptomycin, and mouse NGF (100 ng/mL) for 24 h.

All animal procedures were conducted in accordance with the guidelines of the local animal protection authorities (local district government, Hanover, Germany) and were approved by the relevant authorities. Moreover, the animal studies are in accordance with the ARRIVE guidelines [25] and align with the recommendations set out in the International Journal of Molecular Sciences.

### 4.4. Patch Clamp

Whole-cell patch clamp was performed on HEK 293T cells expressing different constructs of human TRPV1 and human TRPA1. The EPC10 USB HEKA amplifier (HEKA Elektronik, Lambrecht, Germany) was used to low-pass signals at 1 kHz and to sample them at 2 to 10 kHz. Patch pipettes were extracted from borosilicate glass tubes (TW150F-3; World Precision Instruments, Berlin, Germany) to yield a resistance of 2.0–5.0 MΩ. All experiments were conducted at room temperature. Cells were held at −60 mV. Only cells with a high-resistance seal (>1 GΩ) and an initial access resistance <10 MOhm were used for experiments. The external solution contained the following (in mM): NaCl 140, KCl 5, MgCl_2_ 2, EGTA 5, HEPES 10, and glucose 10 (pH 7.4 was adjusted with NaOH). In order to prevent desensitization, calcium was removed from the process. The standard pipette solution (internal solution) contained the following (in mM): KCl 140, MgCl_2_ 2, EGTA 5, and HEPES 10 (pH 7.4 was adjusted with KOH). All solutions were applied using a gravity-driven glass multi-barrel perfusion system. For the heat experiments, the solution was heated from room temperature to approximately 45 °C within 10 s using voltage applied to an insulated copper wire around the capillary tip of the output connection of the perfusion system [26]. This was controlled by Patchmaster software v20x60 software (HEKA Elektronik, Lambrecht, Germany). A miniature thermocouple was attached to the capillary tip to facilitate accurate temperature monitoring. This was positioned in close proximity to the cells under investigation. Voltage ramps were recorded during 500-millisecond intervals from −100 mV to +100 mV. The data acquisition and offline analyses were carried out using Patchmaster/Fitmaster (HEKA Elektronik, Lambrecht, Germany) and Origin 8.5.1 (Origin Lab, Northampton, MA, USA) software.

### 4.5. Illumination

A fluorescent light source (HXP 120, LEJ Lightning & Electronics Jena, Jena, Germany) was used in combination with a filter set consisting of a 340 and 380 nm exciter and a 400 nm dichroic long pass filter (Chroma ET 79001, Chroma Technology GmbH, Olching, Germany). This was used to illuminate the cells with UVA light. It should be noted that the effective intensity of the applied UVA light was not measured.

### 4.6. Ratiometric [Ca^2+^]_i_

The cells were seeded on coverslips a minimum of four hours prior to the commencement of the measurement. Thereafter, they were subjected to staining for approximately 45 min with 4 µM Fura-2-AM and 0.02% pluronic F-127. Following the subsequent washout procedure, the cells were transferred to coverslips, which were then mounted on an inverted microscope (Axio Observer D1, Zeiss, Jena, Germany). Cells were prepared with Fura-2-AM and subsequently illuminated with UV light at 340 nm and 380 nm. The light source used was the HXP120 (produced by LEJ Lightning & Electronics, Jena, Germany), and the LEP filter wheel (manufactured by Ludl Electronic Products Ltd., Hawthorne, NY, USA) was employed in conjunction with appropriate filter sets (produced by Chroma Technology GmbH, Olching, Germany). The images were acquired at a frequency of 1 Hz and exposed for 40 ms using a CCD camera (Cool SNAP EZ, Photometrics, Puchheim, Germany). The data were recorded using VisiView 2.1.1 software (Visitron Systems GmbH, Puchheim, Germany). The standard solution comprised the following mM concentrations of compounds: NaCl: 145; KCl: 5; CaCl_2_: 1.25; MgCl_2_: 1; Glucose: 10; and HEPES: 10. The pH of the solution was adjusted to 7.4. Functional expression of hTRPV1 was verified by application of capsaicin, and the expression of hTRPA1 was verified by application of carvacrol. Cells which did not exhibit calcium influx following application of these agonists were excluded from the analysis. The results obtained are expressed as the mean value (±S.E.M.) of the ratio F340/380 nm.

### 4.7. Western Blot Analysis

Total protein lysates were prepared from HEK293 cells stably expressing TRPA1 and HEK293T cells transiently expressing TRPA1 or TRPV1. Proteins were extracted using RIPA buffer (150 mM NaCl, 1% Nonidet P-40, 0.1% SDS, 0.5% sodium deoxycholate, 50 mM Tris-HCl, pH 7.4) supplemented with protease and phosphatase inhibitors (Roche, Mannheim, Germany).

Protein concentrations were measured, and 10 μg of each lysate was resolved by SDS-PAGE and transferred onto PVDF membranes. Membranes were blocked for 1 h at room temperature with RotiBlock (Roth, Germany) and incubated overnight at 4 °C with primary antibodies against TRPA1([EPR26211-139] from Abcam (Cambridge, United Kingdom) 1:1000 dilution) and TRPV1 (PA1-748, from Thermo Fisher Scientific (Waltham, MA, USA), 1:1000 dilution). Detection was performed using HRP-conjugated rabbit IgG (DakoCytomation, Glostrup, Denmark). Protein bands were visualized via chemiluminescence (SuperSignal West Pico Plus or Femto, Thermo Scientific, Rockford, IL, USA) and captured with a chemiluminescence camera (INTAS, Göttingen, Germany). To confirm equal loading, membranes were stained with Coomassie Blue (0.5% R250, 40% methanol, 10% acetic acid)

### 4.8. Statistical Analysis

The data are presented as mean ± standard error of the mean (S.E.M.), with sample sizes representing the number of measured cells. Imaging data were collected from at least three independent experiments conducted on different days. Patch clamp data were collected from experiments performed on at least two separate days. Related group comparisons used the paired *t*-test, while unrelated groups used the unpaired *t*-test. For comparisons of more than two groups, ANOVA with Tukey’s HSD post hoc test was used. All calculations were performed using Origin 2024b software (OriginLab, Northampton, MA, USA).

### 4.9. Generative Artificial Intelligence (GenAI)

The authors acknowledge that generative AI and AI-assisted technologies, including DeepL Write, DeepL Pro (Version: 25.12.1.19303+a07d1aa03cecbaf3510e79ba54a594de1d12c757) and Academic Cloud (Modell: MetaLlama 3.1 8B Instruct), were utilized to enhance the language and readability of this work. However, the authors have reviewed and edited the content to ensure its accuracy and take full responsibility for the final publication.

## 5. Conclusions

In conclusion, our study demonstrates that staurosporine and midostaurin, two protein kinase inhibitors, exhibit photosensitizing properties that sensitize TRPV1 and TRPA1 channels. Both staurosporine and midostaurin induce a ROS-dependent activation of these channels, with midostaurin also activating TRPA1-expressing mouse dorsal root ganglion (DRG) neurons. These findings are consistent with case reports of phototoxic side effects observed in clinical practice with midostaurin and highlight the potential for photosensitization to contribute to adverse reactions in patients treated with this drug. Our results suggest that the photosensitizing properties of staurosporine and midostaurin may be relevant for understanding their potential phototoxic side effects, and emphasize the importance of considering ROS-dependent mechanisms in the development of phototoxicity.

## Data Availability

The raw data supporting the conclusions of this article will be made available by the corresponding author on request.

## Abbreviations

The following abbreviations are used in this manuscript:

ABCG2	ATP-binding cassette transporter 2
AITC	Allylisothiocyanat
AML	Acute Myeloid Leukemia
ATP	Adenosine Triphosphate
BCTC	4-(3-Chloro-2-pyridinyl)-N-[4-(1,1-dimethylethyl)phenyl]-1-piperazinecarboxamide
Cl^−^	Chloride
DMEM	Dulbecco’s modified Eagle medium
DMSO	Dimethylsulfoxide
DRG	Dorsal Root Ganglion
DTT	Dithiothreitol
EC_50_	Half maximal effective concentration
Fura-2-AM	Fura-2-pentakis-(acetoxymethyl)-ester
HaCaT	Human immortalized keratinocyte cell line
HEK293	Human Embryonic Kidney 293 cell line
K^+^	Potassium
PKC	Protein Kinase C
ROS	Reactive Oxygen Species
TRP	Transient Receptor Potential
TRPA1	Transient Receptor Potential Ankyrin 1
TRPV1	Transient Receptor Potential Vanilloid 1
UV light	Ultraviolet Light
UVA light	Ultraviolet A Light
